# Findings from recent studies by the Japan Aerospace Exploration Agency examining musculoskeletal atrophy in space and on Earth

**DOI:** 10.1038/s41526-021-00145-9

**Published:** 2021-05-26

**Authors:** Satoshi Furukawa, Masahiro Chatani, Atsushi Higashitani, Akira Higashibata, Fuminori Kawano, Takeshi Nikawa, Takuro Numaga-Tomita, Toshihiko Ogura, Fuminori Sato, Atsuko Sehara-Fujisawa, Masahiro Shinohara, Toru Shimazu, Satoru Takahashi, Haruko Watanabe-Takano

**Affiliations:** 1 Human Spaceflight Technology Directorate, Japan Aerospace Exploration Agency, Tsukuba, Ibaraki, Japan; 2 Department of Pharmacology, Showa University School of Dentistry, Tokyo, Japan; 3 Pharmacological Research Center, Showa University, Tokyo, Japan; 4 Graduate School of Life Sciences, Tohoku University, Sendai, Japan; 5 Graduate School of Health Sciences, Matsumoto University, Matsumoto, Nagano, Japan; 6 Department of Nutritional Physiology, Institute of Medical Nutrition, Tokushima University Graduate School, Tokushima, Japan; 7 Department of Molecular Pharmacology, School of Medicine, Shinshu University, Matsumoto, Nagano, Japan; 8 Department of Developmental Neurobiology, Institute of Development, Aging and Cancer, Tohoku University, Sendai, Japan; 9 Department of Growth Regulation, Institute for Frontier Life and Medical Sciences, Kyoto University, Kyoto, Japan; 10 Department of Rehabilitation for the Movement Functions, Research Institute, National Rehabilitation Center for Persons with Disabilities, Tokorozawa, Saitama, Japan; 11 Japan Space Forum, Tokyo, Japan; 12 Department of Anatomy and Embryology, Faculty of Medicine, University of Tsukuba, Tsukuba, Ibaraki, Japan; 13 Department of Cell Biology, National Cerebral and Cardiovascular Center, Suita, Osaka, Japan

**Keywords:** Molecular medicine, Cell biology

## Abstract

The musculoskeletal system provides the body with correct posture, support, stability, and mobility. It is composed of the bones, muscles, cartilage, tendons, ligaments, joints, and other connective tissues. Without effective countermeasures, prolonged spaceflight under microgravity results in marked muscle and bone atrophy. The molecular and physiological mechanisms of this atrophy under unloaded conditions are gradually being revealed through spaceflight experiments conducted by the Japan Aerospace Exploration Agency using a variety of model organisms, including both aquatic and terrestrial animals, and terrestrial experiments conducted under the Living in Space project of the Japan Ministry of Education, Culture, Sports, Science, and Technology. Increasing our knowledge in this field will lead not only to an understanding of how to prevent muscle and bone atrophy in humans undergoing long-term space voyages but also to an understanding of countermeasures against age-related locomotive syndrome in the elderly.

## Introduction

Almost 60 years have passed since Yuri Gagarin became the first human to fly in outer space. Since then, the duration of space missions has increased, and nowadays it is common for astronauts to spend more than 6 months in outer space on the International Space Station (ISS). In addition, plans are underway to build a gateway to the moon and even to go to Mars, which will both necessitate long-term spaceflight missions^1^. However, the microgravity of space is a challenge because it induces bone loss and muscle atrophy in astronauts^2^. This atrophy is problematic because it not only reduces astronauts’ motor function, impairing their ability to perform daily activities once they return to Earth, but it is also associated with negative changes in the cardiovascular system^3^. Interestingly, relationships between spaceflight-induced and aging-induced deconditioning have been suggested^4–6^. For example, there is a common problem of orthostatic intolerance in astronauts after spaceflight and in the elderly, because muscle atrophy weakens venous return to the heart^4,5,7^. Thus, countermeasures developed for use during spaceflight by astronauts may also be useful for the maintenance of elderly health on Earth.

Bone and skeletal muscle are dynamic tissues that undergo continuous synthesis and degradation. Bone remodeling is performed by bone-forming cells called osteoblasts and bone-resorbing cells called osteoclasts^8^, and maintenance of skeletal muscle mass is the result of a balance between cellular protein synthesis and catabolism^9^. Bone and skeletal muscle mass can be altered in response to increased loading with exercise or to decreased loading with spaceflight or disuse, which is summarized as the “use it or lose it” concept of bone adaptation known as Wolff’s Law^10^. Various experiments examining the effects of long-term spaceflight on the musculoskeletal system have been conducted by the United States and Russia using space shuttles, unmanned satellites, and the ISS. The findings from these experiments have provided insights into how the muscles are changed during deloading. For example, analysis of the skeletal muscle of mice exposed to microgravity has revealed that muscle atrophy occurs mainly in the anti-gravity muscles such as the soleus muscle. Microgravity-induced atrophy is accompanied by an increase in the ratio of fast-twitch to slow-twitch muscle fibers^11^.

The Japan Aerospace Exploration Agency (JAXA) has conducted several spaceflight experiments to study the effects of space environments on muscles and bones using the Space Shuttle, Soyuz, SpaceX, and the ISS. As an example of a pioneering JAXA experiment conducted during mission STS-90 of the Space Shuttle *Columbia* in 1998, Ikemoto et al. demonstrated increased degradation of fast-type myosin heavy chain protein and increased protease expression in atrophied gastrocnemius muscles of neonatal rats exposed to 16 days of spaceflight; spaceflight was also found to stimulate the ubiquitination of proteins such as myosin heavy chain, and the accumulation of myosin heavy chain degradation fragments in muscle^12^. From 2008 to 2009, the Japanese Experiment Module (JEM), which is a part of the ISS, was assembled to create a laboratory called “Kibo” on the ISS^13^. After that, Kibo was equipped with cell biology experiment facilities (CBEF and CBEF-L)^14^ and a fluorescent microscope, Confocal Space Microscopy (COSMIC)^15^. JAXA also developed an aquatic habitat (AQH)^16^ for aquatic animals, a system for housing mice (Mouse Habitat Unit: MHU)^17^ for use in space experiments that includes live imaging and an artificial gravity centrifuge and a multiple artificial-gravity research system (MARS) for rodents^18^. In particular, MHU and MARS can create artificial gravity to properly study the effects of differences in gravity. These unique systems will allow scientists to investigate the effects of gravity differences on rodents thoroughly.

Although these spaceflight experiments have afforded a wealth of insights and novel findings, further studies are needed to fully understand the molecular mechanisms of bone loss and muscle atrophy during spaceflight and to determine the preventative effects of exercise and other potential countermeasures. On the ISS, crewmembers are scheduled 2.5 h of exercise a day, 6 days a week, to prevent bone loss and muscle atrophy^19^. However, the astronauts still lose an average of 3% of their bone mineral density at the lumbar spine and 6% at the hip and pelvis after the typical 6-month missions^20^. Furthermore, a marked variation has been observed among individuals, with some astronauts losing up to 10–15% of their bone mineral density, which is equivalent to a loss of 1.7–2.5% per month during a 6-month mission^20^. This large variation in the effects of exercise is likely the result of a variety of factors, including genetic background, exercise compliance, exercise intensity, and exercise dynamics.

The next challenges for humankind include new missions to the Moon, followed by human exploration to Mars. For the missions to Mars, the long travel distance will make the total mission duration 800–1100 days, of which approximately 500 days is expected to be spent on the planet’s surface^21^. As a result, astronauts will spend much more time under micro-gravity or low-gravity compared with a mission on the ISS. Thus, one of the major concerns for such long space missions is astronaut health with respect to bone loss and muscle atrophy. In this review, we summarize the musculoskeletal findings of recent terrestrial experiments conducted as part of the Living in Space project of the Japanese Ministry of Education, Culture, Sports, Science and Technology, and of spaceflight experiments conducted in the “Kibo” Japanese Experiment Module of the ISS.

## Recent JAXA space missions related to muscle and bone

Since 1998, JAXA has conducted 14 space missions onboard the ISS to understand the causes of muscle atrophy and osteopenia under microgravity environments (Table 1). So far, cultured cells, *Caenorhabditis elegans*, medaka, goldfish scales, zebrafish, mice, and humans have been the subjects of these experiments. It is expected that the genetic, molecular, tissue and behavioral data accumulated under different gravitational conditions will allow elucidation of the mechanisms underlying homeostasis of the musculoskeletal system. Here, we summarize the findings of the most recent animal experiments conducted by JAXA in the Japanese Experiment Module of the ISS.

**Table 1:** JAXA space missions related to musculoskeletal research.

Year	Transportation	Subject	Research target	Publications
1998	Space shuttle *Columbia* (STS-90)	Rats	Muscle	Ikemoto M. et al.^12^, Nikawa T. et al.^24^
2010	Space shuttle *Discovery* (STS-131), ISS	Rat cells (myotubes)	Muscle	Uchida T. et al.^22^
2004	Soyuz TMA-4, ISS	*C. elegans*	Muscle	Higashibata A. et al.^33,34^, Selch F. et al.^35^
2009	Space shuttle *Atlantis* (STS-129), ISS	*C. elegans*	Muscle	Etheridge T. et al.^36^, Higashibata A. et al.^37^, Harada et al.^38^
1994	Space shuttle *Columbia* (STS-65)	Medaka	Ontogeny	Ijiri K. et al.^41^
2012	Soyuz TMA-06M, ISS	Medaka	Bone	Chatani M. et al.^42^
2014	Soyuz-U, Progress M-22M (54P), ISS	Medaka	Bone	Chatani M. et al.^43^
2010	Space shuttle *Atlantis* (STS-132), ISS	Goldfish scales	Scales	Ikegame M. et al.^47^
2014	Soyuz TMA-14M, ISS	Zebrafish	Muscle	Sato F. et al., in preparation
2018	SpaceX Falcon 9, ISS	Zebrafish	Muscle	Sato F. et al., in preparation
2016	SpaceX Falcon 9, Dragon, ISS	Mice	Bone, muscle	Shiba D. et al.^18^
2017	SpaceX Falcon 9, Dragon, ISS	Mice	Bone	Tominari T. et al.^52^
–*	ISS	Humans	Bone, bisphosphonate	Leblanc A. et al.^100^ Sibonga J. et al.^101^

*Ethical considerations.

### Cell culture experiments

**Fig. 1:**
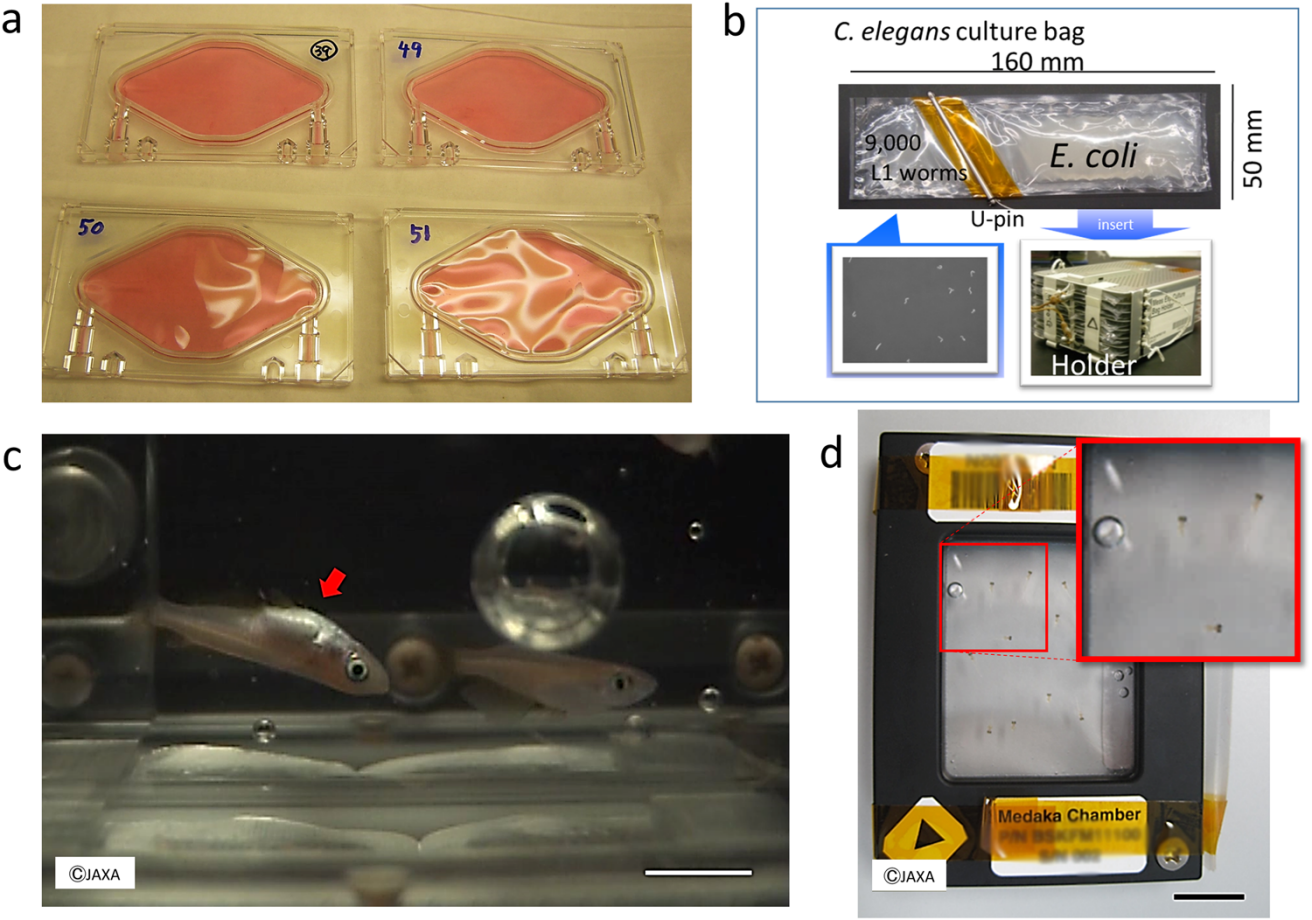
Recent JAXA space missions in Kibo of ISS. **a** Cell culture system used in the Myo Lab project^22^. L6 rat myoblasts at 100% confluence in a disposable cultivation chamber (DCC) were fused by changing the medium to a differentiation medium (i.e., Dulbecco’s modified Eagle’s medium containing 0.5% fetal bovine serum). (Photo by Nikawa, T) **b** *C. elegans* culture system used in the CERISE project^36–38^. Upon reaching microgravity at KIBO, flight crews activated the experiments by removing the U-pin. Holder was set into Meas Exp Unit A and transferred into the Cell Biology Experiment Facility (CBEF) with or without 1-*g*  rotation for either 4 or 8 days. (Photo by Higashitani, A). **c** Appearance of medaka reared during spaceflight. Medaka was filmed for abnormal behavior to examine physiological changes under microgravity. The recordings showed that the fish became accustomed to life under microgravity but displayed unique behavior such as swimming upside-down (red arrow). Scale bar, 10 mm. **d** Medaka chamber for live imaging used on the ISS. Twelve larvae at stage 39 can be kept in each “Medaka Chamber”, in which the larvae are placed in Mebiol Gel (Mebiol Inc., Kanagawa, Japan) and then covered with a gas-permeable membrane. The chamber shown was carried aboard *Soyuz* flight progress M-22M (54 P) (Roscosmos, Russia) in 2014. After arrival at the Japanese Experiment Module of the ISS, the chambers were set under a fluorescence microscope for live imaging for 8 days. The red dotted area indicates an enlarged view (inset) showing three larvae. Scale bar, 10 mm.

In 2010, as part of space mission STS-131, JAXA conducted an experiment to examine muscle atrophy-associated ubiquitin ligase casitas B-lineage lymphoma-b (Cbl-b) in rat myoblasts^22^. In this experiment, L6 rat myoblasts at 100% confluence were allowed to differentiate for 5 days in a differentiation medium and were then sent to the ISS (Fig. 1a). Seven days after launch, the culture medium was replaced with fresh DMEM with or without 40 ng/mL insulin-like growth factor-1, which induces skeletal myocyte hypertrophy, and the cells were cultured for 10 days under microgravity or 1-*g* conditions, the latter achieved by an artificial gravity centrifuge in KIBO. In myoblasts and myotubes, gravitational unloading (with 3D clinorotation) resulted in a rapid increase in the production of reactive oxygen species (ROS), which are critical upstream mediators that link downstream atrophic signaling with metabolic changes^23^. Flow cytometry with nitric oxide (NO)-specific fluorescence indicator failed to detect NO after clinorotation in myoblast, indicating that unloading stress did not increase NO radicals. Administration of *N*-acetylcysteine or TEMPOL (1-oxyl-2,2,6,6-tetramethyl-4-hydroxypiperidine) to L6 myotubes significantly suppressed clinorotation-mediated activation of extracellular signal-regulated kinase (ERK) 1/2; however, the suppressive effect of TEMPOL on clinorotation-mediated ERK1/2 activation was stronger than that of *N*-acetylcysteine. In addition, because TEMPOL mimics superoxide dismutase that catalyzes the dismutation of the superoxide radicals^23^, microgravity and clinorotation resulted in the production of superoxide anions.

The generation of ROS was found to activate two signaling pathways in the myotubes: (1) the ERK 1/2-early growth response protein (Egr) 1/2-Cbl-b signaling pathway, which is involved in muscle volume loss, and (2) the mitochondrial aconitase-associated dynamin-related protein 1 signaling pathway, which is a regulator of mitochondrial organization and function. The first pathway was found to upregulate Cbl-b via an early growth response protein consensus element at base pairs −110 to −60 of the Cbl-b promoter, resulting in a potent oxidative stress response. This suggests that under microgravity, elevated levels of reactive oxygen species in myotubes may be crucial mechanotransducers that regulate muscle mass via expression of the ubiquitin ligase Cbl-b. Previous experiments in Cbl-b-deficient mice have shown that Cbl-b plays an important role in not only microgravity-induced skeletal muscle atrophy but also in atrophy induced by bed-rest^24,25^. The unique mechanism of this Cbl-b-mediated muscle atrophy does not appear to involve the degradation of structural muscle components; rather, it seems that Cbl-b impairs muscular trophic signals in response to unloading conditions. Indeed, ubiquitinated Cbl-b has been shown to induce specific degradation of insulin receptor substrate 1, a key signaling molecule in skeletal muscle growth, resulting in downregulation of insulin-like growth factor-1 signaling^22,24,25^.

Furthermore, at the genetic level, spaceflight has been shown to significantly increase the mRNA levels of several muscle atrophy-associated ubiquitin ligases (E3), including muscle RING-finger protein-1 (MuRF-1), muscle atrophy F-box-1 (MAFbx-1)/atrogin-1, and Cbl-b^24,26^. In 2014, the first-ever spaceflight experiment using gene-knockout mice was conducted using MuRF1-deficient mice^27^. Under microgravity, these mice showed muscle atrophy comparable with that of wild-type mice, suggesting that a number of redundant systems are involved in microgravity-induced muscular degradation. Other proteolytic pathways are also implicated in atrophy under spaceflight, including calpain-mediated pathways, apoptosis, and matrix metalloproteinase-mediated pathways^28,29^. In addition, downregulation of insulin-like growth factor-1-signaling mediated MAFbx-1/atrogin-1 expression through dephosphorylation (activation) of FOXO3 has been shown to reduce the skeletal muscle mitogen response^30,31^. This is consistent with previous observations of suppression of the Akt-mTOR anabolic pathways under microgravity, suggesting that microgravity affects a complex pathway that involves both anabolic and proteolytic systems^30–32^.

### Caenorhabditis elegans experiments

Model organisms, such as the nematode *Caenorhabditis elegans*, are useful to understand the effects of microgravity. *Caenorhabditis elegans* is particularly suitable for spaceflight experiments because they are easy to culture and require little space, there is a rich suite of genetic and molecular tools with which they can be examined, and they have a common molecular basis for human and rodent modeling. In 2004, the “International *C. elegans* Experiment—First in Space” experiment (ICE-FIRST) was carried out by an international collaboration of laboratories. Transcriptome analysis of *C. elegans* after 10 days of spaceflight revealed alteration of the expression of muscle-related genes, as well as genes that are regulated by insulin and transforming growth factor-β signaling^33–35^.

In 2009, the *C. elegans* RNA Interference Space Experiment (CERISE) was conducted in which nematodes in a liquid medium with food comprising bacteria expressing target double-stranded RNA were cultured for 4 days under microgravity or 1-*g* conditions (Fig. 1b). Upon return to Earth, RNA interference activity, and global gene and protein expression, were analyzed using DNA microarray and mass spectrometry techniques^36,37^. Gene silencing for ectopically expressed green fluorescent protein and endogenously expressed genes encoding the RING finger protein RBX-1 for some complexes of E3 ubiquitin ligases and aspartic proteinases ASP-4/6 occurred normally under microgravity^38^. Expression levels of gene and protein in muscular thick filaments were also significantly reduced in *C. elegans* grown from the L1 larval stage to adulthood under microgravity compared with those grown under 1*-g* conditions^37^. In addition, the expression of cytoskeletal elements and mitochondrial metabolic enzymes were decreased by microgravity and decreased locomotor activity (thrashing rate), body length, and fat accumulation were observed in the nematodes cultured under microgravity compared with those cultured under 1*-g* conditions^37^. Also, similar reductions in the expression of genes such as a myosin heavy chain 3 and a transforming growth factor-β gene, *dbl-1*, were observed in nematodes exposed to microgravity and those cultured under decreased fluid dynamic conditions (i.e., reduced viscosity/drag resistance or reduced depth of the liquid culture)^38^. *dbl-1* is a positive regulator of macromolecule biosynthetic processes such as collagens, c-type lectin, mucin, catecholamine, and lysozymes, and an ortholog of mammalian bone morphogenetic protein 10, which is essential for maintaining cardiac growth and function^39,40^. These results suggest that there is a common mechanism between *C. elegans* and humans that reduces muscle development and maintenance in response to microgravity. These findings support the use of *C. elegans* in future mechanistic studies to examine how microgravity impacts muscle mass, metabolism, and growth factor expression in humans. In addition, future space experiments are awaited for the analysis of mechanisms and tissues related to gravitational effects, mechanical stimuli, contact stimuli, and these responses using the nematode *C. elegans*.

### Medaka, goldfish scale, and zebrafish experiments

Medaka (*Oryzias latipes*) is a small freshwater teleost fish with properties suitable for studies in space, including a short generation time, compact size, and translucent body until the larval stage. Medaka was first sent to space in 1994 and was successfully mated under microgravity^41^. In 2012, medaka was raised for approximately 2 months in the Aquatic Habitat system onboard the ISS (Fig. 1c) and examinations revealed the reduced mineral density of the pharyngeal teeth and bone, as well as osteoclast activation^42^. In addition, electron microscopy revealed that the mitochondria of the osteoclasts were less circular. The whole transcriptome analysis showed that *fkbp5* and *ddit4* genes, known as the downstream of the glucocorticoid signaling, were strongly upregulated in the flight group reared for 60 days at ISS^42^. Furthermore, the fish were filmed for abnormal behavior such as swimming upside-down, and it was found that the medaka tended to become motionless in the late stage of exposure. Considering that all of the medaka remained healthy over the 2-month period, it can be inferred that the fish changed their behavior in response to the microgravity environment. Together, these findings confirmed that medaka is an acceptable animal model for the analysis of biological responses to altered gravity.

**Fig. 2:**
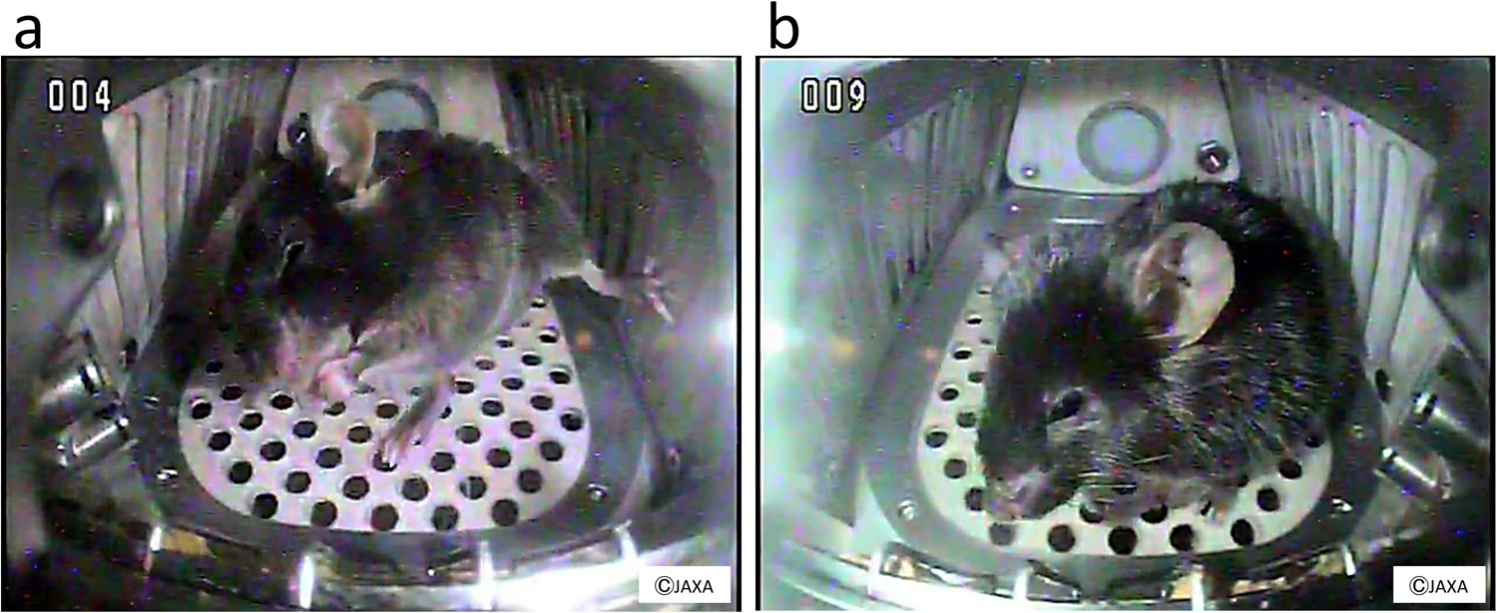
Mice in space. **a** A still image captured from a video recording of a C57BL/6J male mouse in microgravity in the ISS. **b** A still image captured from a video recording of a C57BL/6J male mouse under artificial 1 *g* conditions generated by a short-arm centrifuge in the ISS. These mice were housed in the mouse habitat cage unit for 35 days and these images were taken on day 5.

To examine the initial action of microgravity, another space experiment was conducted in 2014. The osteoblast and osteoclast-specific transgenic medaka larvae housed in special chambers and sent to the ISS (Fig. 1d). In live imaging for osteoblasts, the intensity of *osterix*-DsRed or *osteocalcin*-DsRed fluorescence in pharyngeal bones was significantly enhanced 1 day after launch. In osteoclasts, the signals of *TRAP*-GFP and *MMP9*-DsRed were highly increased at days 4 and 6 after launch in flight. HiSeq analysis from pharyngeal bones of juvenile fish at day 2 after launch showed significant changes in genes related to glucocorticoid signaling^43^. This experiment revealed that exposure to microgravity immediately induced a change in gene expression levels in osteoblasts and osteoclasts.

In a different experiment examining the participation of osteoblasts and osteoclasts in bone healing, *osterix*-DsRed/*TRAP*-EGFP double-transgenic medaka was treated with synthetic glucocorticoid, prednisolone. At 18 days after initiation of continuous prednisolone administration, a part of the bony fin ray was fractured. Bone fracture healing was significantly delayed by up to 32 days and was accompanied by decreased osteoblast and osteoclast signaling as compared with control fish. To confirm the function of glucocorticoid receptors in bone healing, a glucocorticoid receptor 2-deficient (*gr2*^−/−^) medaka line was constructed. The results showed that *osterix*-DsRed and *TRAP*-EGFP fluorescent signals were increased at the site of the bone fracture in those fish. These results demonstrated negative regulation of osteoclast recruitment by glucocorticoid receptors and accumulation of osteoblasts during bone fracture healing^44^. It is true that there is a change in a part of glucocorticoid signaling in space, but it is inferred that the phenomenon is slightly different from that of glucocorticoid administration. It is expected to be clarified by detailed analysis in the future.

Using a centrifuge designed for small-fish rearing, the effect of hypergravity on medaka reared for 6 months under normal gravity (1 *g*) or under gravity, approximately 5 times normal (5 *g*) was investigated^45^. Micro-computer tomography analysis revealed that although the fish were able to maintain body posture and position, hypergravity gradually induced vertebral curvature towards the dorsal side and asymmetric formation of otoliths in which the cross-sectional area was increased. These findings indicate that the process of adaptation to a hypergravity environment results in spinal deformation and otolith abnormality in medaka. Together, these experiments confirm the potential of using medaka to elucidate the detailed mechanisms that underlie responses to altered gravity.

To examine the effects of melatonin, which has been reported to inhibit osteoclast function under microgravity conditions^46^, scales of goldfish were used as a model of coexisting osteoclasts and osteoblasts in the experiment conducted on the ISS. Microgravity was found to stimulate osteoclast activity and significantly increase the expression of genes involved in osteoclast differentiation and activation. Melatonin treatment significantly increased expression of calcitonin (an osteoclast-inhibiting hormone) mRNA but decreased the mRNA expression of receptor activator of nuclear factor kappa-B ligand (RANKL: a promoter of osteoclastogenesis), and these changes coincided with suppression of the expression of genes associated with osteoclastogenesis. The mRNA expression level of acetylserotonin *O*-methyltransferase, an enzyme essential for melatonin synthesis, was significantly decreased under microgravity^47^. This was the original study to suggest an inhibitory effect of melatonin on osteoclast activation by microgravity.

To examine the mechanisms of terrestrial age-related muscle atrophy, an experimental system to investigate muscle atrophy in zebrafish was developed. RNA-seq analyses revealed significant increases in the amounts of transcripts related to the proteasome system and to autophagy in skeletal muscle when the mobility of zebrafish was decreased. To further elucidate the general mechanisms of muscle atrophy, experiments on muscle atrophy that occurs during spaceflight in 2014 were conducted. The aim of this project (named Zebrafish Muscle) was to examine whether skeletal muscle atrophy occurs in adult zebrafish under microgravity^48^. Zebrafish were bred on the ISS for 6 weeks and live monitoring revealed that the fish learned to swim under microgravity much quicker than expected^49^. To understand the mechanisms of skeletal muscle atrophy in space, transcriptome data were periodically obtained from the skeletal muscle of the fish during their stay in space and during their recovery after return to Earth; it is expected that analysis of this data will allow us to understand further the process of skeletal muscle atrophy in space and the role of gravity in the maintenance and homeostasis of skeletal muscle on Earth.

### Mouse experiments

A significant decrease in the mass of weight-bearing bone, but not of non-weight-bearing bone, has been observed in space-flown mice^50–52^. Osteoclast activation may be one mechanism of this bone loss;^53^ however, further analyses are needed to understand the mechanistic details. It has also been reported that the signaling involved in this bone loss is compartmentalized to discrete cells (e.g., osteoclasts) and signaling pathways^54^. For example, RANKL has been shown to activate NADPH oxidases (e.g., Nox2), which produce ROS (primarily H_2_O_2_) that induce osteoclast proliferation and activation, as observed in osteoporosis^55^. Thus, it is possible that similar signaling is involved in skeletal muscle atrophy under microgravity^56^.

In 2016, a 35-day space experiment in mice was conducted using an artificial gravity generator developed by JAXA (Fig. 2). As in previous space experiments using mice, bone loss and muscle atrophy were observed; however, the artificial 1-*g* condition completely suppressed these changes^18^. It is expected that this system will allow more detailed analyses of the effects of the space environment on mice to be performed in the future since partial gravity can be used for quantitative analysis. Transcriptome analysis of the soleus muscle of space-flown mice is currently underway to study the effects of microgravity on skeletal muscle atrophy. Preliminary data show that the expression level of certain atrophy-related genes changes in the soleus of mice under microgravity but the myostatin-encoding gene does not change compared with in-ground controls or artificial 1-*g* controls onboard the ISS.

**Fig. 3:**
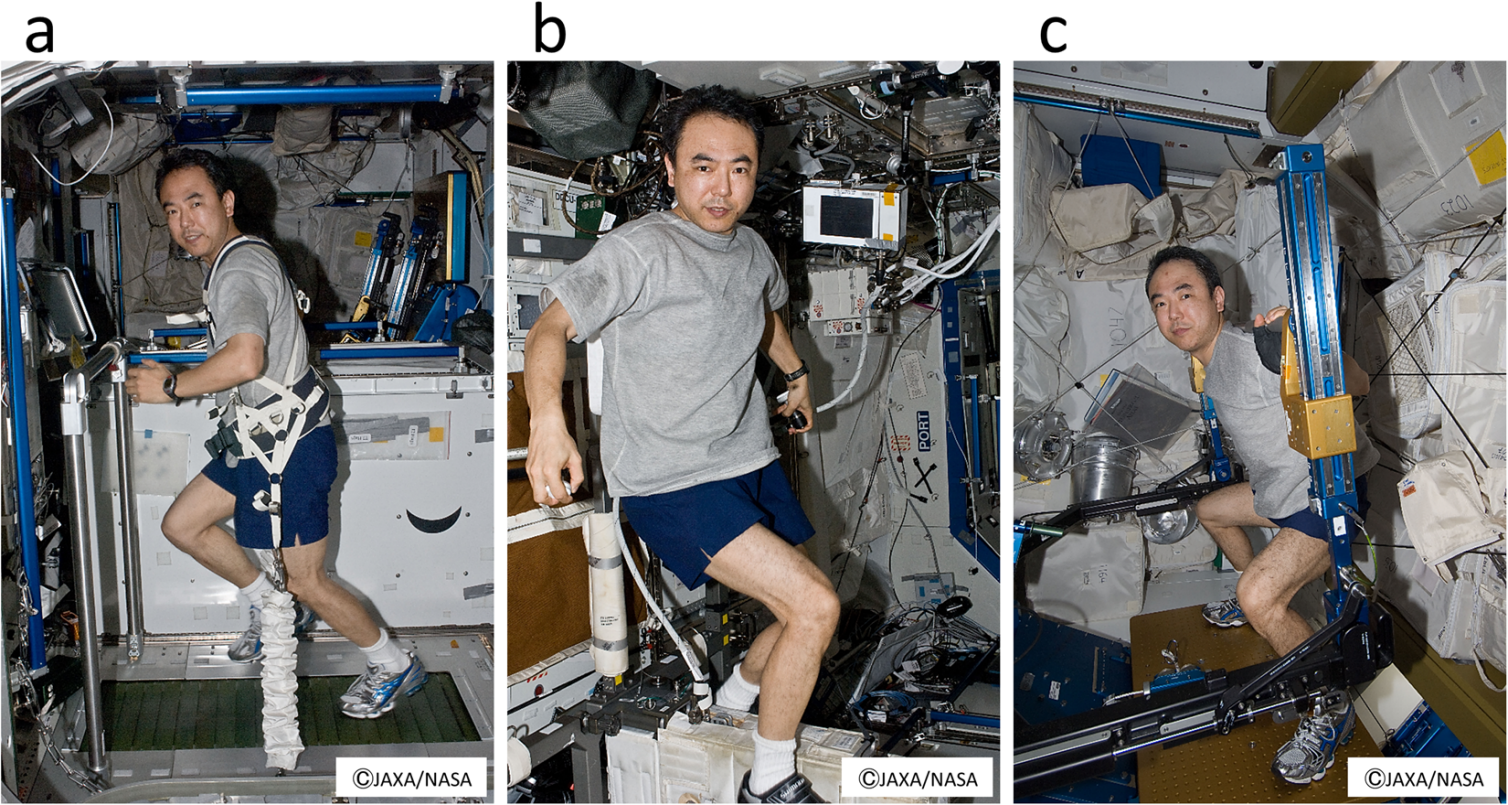
Japanese astronaut Satoshi Furukawa exercising on the T2 treadmill, the Cycle Ergometer with Vibration Isolation and Stabilization System (CEVIS), and the Advanced Resistive Exercise Device (ARED) in the ISS. Astronauts have daily exercise sessions, which are part of operational tasks onboard the ISS, to minimize muscle atrophy and bone loss risks. The first author and an astronaut S.F. agreed and gave written consent to use these photographs. **a** T2 treadmill, (**b**) CEVIS, and (**c**) ARED.

On the other hand, no upregulation of the myostatin gene is observed in the muscles of spaceflown mice. Myostatin is a highly conserved member of the transforming growth factor (TGF)-β superfamily, the members of which are negative regulators of vertebrate skeletal muscle mass. Myostatin inhibitors enhance muscle strength and functional performance against wasting disorders^57^. In addition, increased bone mineral density in the femurs of myostatin-knockout mice has been reported^58^. It was also found that the expression of TGF-β1 (a positive regulator of myostatin^59^) decreased in response to microgravity. This is consistent with in vitro observations in human fetal osteoblastic cells grown in space^60^, in vivo observations in 11-day space-flown rat skeletal muscle^61^, and in vivo observations in colonic tissue and systemic lymph node levels of 91-day space-flown mice^62^. Together, these data suggest that microgravity does not induce muscular atrophy via direct upregulation of the negative regulator myostatin.

One of the cell types responsible for sensing physical loading is the skeletal stem cell (SSC) because loading-induced bone formation requires activation of these cells in the periosteum to give rise to osteoblasts. Alteration of the gene expression pattern and cell morphology within the periosteum after loading suggests that these skeletal stem cells sense loading stimuli and remodeling^63–65^. In addition, mitochondrial function is likely to be affected by microgravity^24,37,66^, and the role of mitochondrial energy production mediated by oxidative phosphorylation in skeletal muscle stem cells (SMSCs) has been investigated. SMSCs are normally cell-cycle arrested but differentiate to generate myocytes upon muscle damage, and along with self-renewing SMSCs form new myofibers. The end product of glycolysis, pyruvate was found to be a substance that stimulates their growth and differentiation^67^. Pyruvate dephosphorylates and activates pyruvate dehydrogenase, which opens the gateway from glycolysis to the tricarboxylic acid cycle by producing acetyl coenzyme A from pyruvate. Conditional deletion of pyruvate dehydrogenase in SMSCs reduces cell division associated with the generation of myocytes and subsequent myotube formation, decreases skeletal muscle regeneration upon injury, and aggravates the pathogenesis of Duchenne muscular dystrophy in dystrophin-deficient mice. These results indicate that the flow from glycolysis to the tricarboxylic acid cycle mediated by pyruvate dehydrogenase plays a critical role in the differentiation of SMSCs^67^ and that decreased oxidative phosphorylation affects SMSC differentiation in space.

## Countermeasures to prevent bone and muscle atrophy under microgravity

### Current exercise program on the ISS

The current exercise program on the ISS consists of two exercise sessions per day (30–45 min of aerobic exercise and 45 min of resistance exercise) and exercise is scheduled 6 days a week^19,68^. Many astronauts work out 7 days a week, although the seventh day is officially considered a rest day. The allotted exercise time includes time for changing clothes, exercise equipment setup and stowing, and post-workout hygiene^19^.

In the United States’ on-orbit segment of the ISS, there are two aerobic devices (a T2 treadmill and a Cycle Ergometer with Vibration Isolation and Stabilization system [CEVIS]) and one resistance device (the Advanced Resistive Exercise Device [ARED]) (Fig. 3). For aerobic exercise, a Treadmill with a Vibration Isolation and Stabilization system was initially used but has now been replaced by a T2 or CEVIS system^19^. Aerobic sessions consist of steady-state and interval-type protocols, with target intensities of 75–80% or 60–90% VO_2_ max^68^. Resistance exercise was initially performed with the interim Resistive Exercise Device but is now performed using the ARED^19^. Resistance protocols are multi-set, multi-repetition for the lower and upper body, with initial loads calculated from a 10-repetition maximum load (plus 75% of body weight to compensate for the absence of body weight) and adjusted thereafter based on actual performance^68^. In future space missions and analog studies, exercise programs could be complemented with lower body negative pressure (LBNP) application^69^ to assess whether this leads to a reduction of post-spaceflight orthostatic intolerance and improvements in general astronaut health, including muscle and cardiovascular health.

**Fig. 4:**
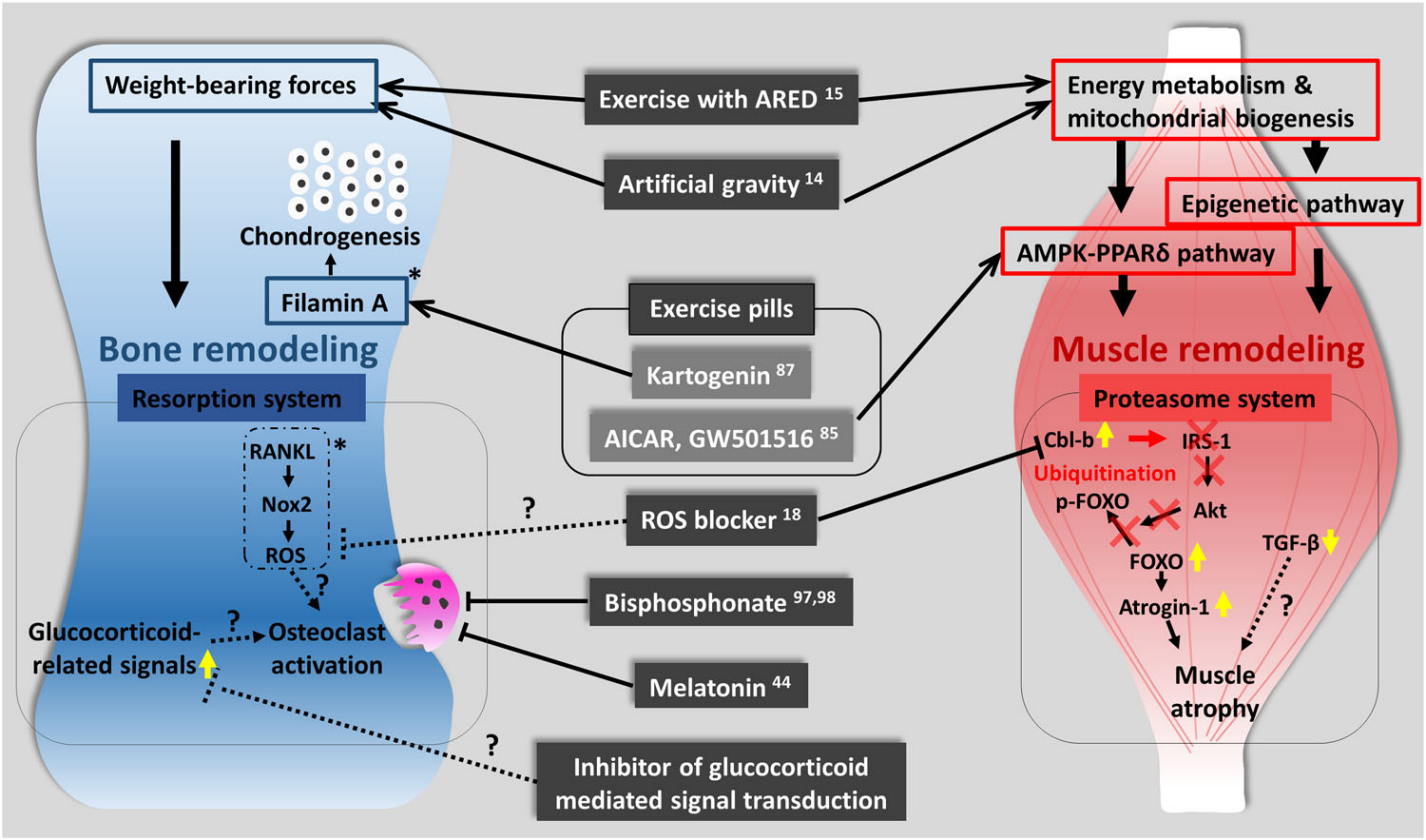
Musculoskeletal atrophy and its countermeasures in space. Key pathways to prevent bone and muscle deterioration in the space environment are represented. Yellow arrows indicate the molecules whose expression changes have been studied in space experiments. Asterisks indicate ground-based experiments and candidates for future space experiments. Question marks indicate candidates for future ground and space research.

### Exercise epigenetics in skeletal muscle

Exercise training during a long-term stay on the ISS has been found to be effective, at least in part, in preventing disuse atrophy in skeletal muscle. A previous study has reported that daily exercise (2.5 h per day) suppressed the decrease of slow-twitch-fiber size in soleus muscle, with the astronauts retaining 67% of the pre-flight level after prolonged spaceflight (approx. 180 days)^70^. However, the exercise results differed depending on the individual, with two of nine crew members exhibiting drastic loss of muscle fiber size (<30% of a pre-flight level) even though they performed the scheduled exercise training during the flight. It is well known that individuals respond differently in terms of muscle mass gain to the same exercise protocol. Therefore, the individual difference in the effects of exercise training is a major issue that must be addressed before longer-term space missions can be undertaken.

It is reported that muscle mass gain after resistance exercise training is positively correlated with the expression level of a particular microRNA, miR-378^71,72^, and also positively correlated with upregulation of mechanical-growth factor and myogenin gene expression^73,74^. DNA methylation in human skeletal muscles is also affected by resistance exercise training^75,76^. Seaborne et al. have reported that the frequency of genome-wide hypomethylation at CpG sites in human skeletal muscle is increased after chronic resistance exercise training, is maintained during a detraining period, and is further enhanced in response to reloading^75^. Furthermore, Turner et al. reported that long-term resistance exercise training upregulated the expression of 592 of 5262 genes that were hypomethylated at their CpG sites^76^. Together, these results suggest that epigenetic regulation results in differences in responsiveness of skeletal muscle to exercise training.

In animal studies, Nakamura et al. have reported that disuse atrophy is suppressed in the fast-twitch skeletal muscles of adult rats with prior experience of running training using a treadmill^77^. They also found that the expression of a subset of genes that are generally upregulated during disuse atrophy in sedentary rats was less responsive to unloading in the fast-twitch fibers of the plantaris muscle of rats with training experience. Canonical histone 3, a main component of nucleosomes, was replaced with the H3.3 variant at these loci after the training period. It has also been reported that these running training-associated exchanges of histone components were induced relative to the amount of exercise^78^. Together, these results indicate that histone component turnover is stimulated by running exercise and that this turnover alters gene responsiveness to later stimuli.

Epigenetic regulation differs between fast-twitch and slow-twitch skeletal muscle fibers. Transcriptionally active histone modifications, such as acetylation and tri-methylation at lysine 4 of histone 3, have been found to be prevalent at loci with higher expression in fast-twitch muscle fibers of adult rats, although no relationship between histone modifications and gene expression was seen in slow-twitch muscle fibers^79^. Furthermore, the expression of slow-twitch fiber-specific genes was upregulated in fast-twitch muscle fibers but, if muscle activity was enhanced by surgery, the level of histone acetylation at the loci was decreased in rats^79^. Masuzawa et al. have reported that the transcriptional activation of peroxisome proliferator-activated receptor-gamma coactivator 1-alpha gene in response to acute running exercise using a treadmill in rats is greater in fast-twitch muscle fibers in which acetylation of histones is more prevalent as compared to that in slow-twitch muscle fibers^80^. A unique regulation system of gene expression in slow-twitch skeletal muscle fibers is suggested to be closely related to tonic neural activity, even in sedentary posture under normal gravity^81,82^.

### Exercise pill

Exercise is one way to prevent sarcopenia and osteoporosis, which are the most frequent symptoms observed in astronauts living under microgravity. Astronauts continue regular exercise in a small training facility built into their spacecraft, but the physical activities performed in this restricted space are insufficient, and they often show ataxy after returning to Earth. To address this issue, novel medical aids must be developed. An exercise pill (exercise mimetic) is a potential pharmaceutical approach that could address this issue in both astronauts and sedentary people on Earth.

Skeletal muscle is highly adaptive to external stimuli, such as mechanical, physiological, and nutritional demands. Exercise remodels the energy metabolism of myofibers by converting type-IIb glycolytic fibers to the more oxidative type-IIa fibers to increase lipid metabolism and to stimulate mitochondrial activity^83^. In the elderly, oxidative type-IIa muscle fibers are more resistant to atrophy^84^ and show higher exercise endurance and fat-burning metabolism; therefore, drugs that promote the formation of oxidative type-IIa muscle fibers are potentially beneficial in combatting lifestyle-related diseases such as obesity and diabetes. Druggable targets for exercise pills could be the key proteins of the regulatory system of this muscle remodeling, although the mechanism underlying how exercise boosts this adaptation is currently unknown^85^.

Recently, several key regulators of this adaptation have been reported, and they in part involve ATP/AMP, NADH/NAD^+^ metabolism, and the metabolic sensors AMP-activated protein kinase (AMPK) and sirtuin 1^86,87^. In addition to these sensing systems, several nuclear receptors, such as estrogen-related receptors and peroxisome proliferator-activated receptors (PPARs), act as key transcriptional regulators in concert with the PPARγ co-activator α/β. Among the three PPARs (α, γ, and β/δ), PPARδ is known to play a pivotal role in fatty acid metabolism of skeletal muscle. Its overexpression in muscle promotes the remodeling of glycolytic myofibers into oxidative ones with higher fatty acid oxidation and mitochondrial biogenesis, resulting in super endurance performance (i.e., the so-called “marathon mice”).

In addition to genetic modification, pharmaceutical approaches have also been reported^88^. Administration of a synthetic ligand of PPARδ, GW501516, activates fatty acid oxidation and energy expenditure in muscle, but when co-administrated with AICAR (acadesine/AICA riboside), a potent AMPK activator, mitochondrial biogenesis, and oxidative metabolism are synergistically boosted, suggesting that the AMPK-PPARδ pathway is a potential target for an exercise pill to improve exercise endurance and fatty acid oxidation. Extensive searches for druggable targets for an exercise pill have been made in the signaling cascades described above. Natural compounds, such as urolithin A, are known to improve exercise capacity in rodents and prevent the age-related decline of muscle function^89^, although the primary protein target of this compound is currently unknown. Similarly, some compounds are known to have anti-aging activities, particularly anti-sarcopenia effects, and the search for novel druggable targets should be expanded to finding targets for the so-called geroprotectors.

Kartogenin (KGN) is a small compound that promotes chondrocyte differentiation, chondroprotection, and cartilage repair^90^. KGN binds filamin A, which disrupts its interaction with the transcription factor CBFβ, and induces chondrogenesis by regulating CBFβ-RUNX1 transcriptional regulation^90^. KGN is a potential lead for formulation into an exercise pill because it is known that moderate exercise causes anabolic responses in chondrocytes and cartilage exhibits increased proteoglycan content, decreased proteoglycan degradation, and increased thickness^91^. In addition, it appears that traditional Chinese medicine may play important role in alleviating muscle and bone loss. Several traditional Chinese medicines are currently being investigated in this regard^30,92^.

Comprehensive studies in this area should pave the way for a more scientific exercise assessment of individuals and the development of safe and effective exercise pills. This will help humans live healthier and safer in microgravity and low-gravity environments for long periods of time without bone loss and muscle atrophy.

## Discussion

### New concepts in the context of muscle atrophy and osteopenia under microgravity

All living organisms are directly exposed to the effects of gravity. In mammals, such as mice and humans, the body must work to overcome gravity and maintain posture. In nematodes and fish, the different body construction compared with that of mammals means the effects of gravity are likely different. This same must also be true for cultured cells and fish scales. While the effects of gravity likely vary from species to species, it is interesting to note that the JAXA space missions have shown in all the subjects examined that muscle and bone tend to become weaker under microgravity. This indicates that it is important for cells, including mammalian cells, to be under the direct effects of gravity. Indeed, it has been reported that the radius of people who have experienced a microgravity environment has unexpectedly developed post-flight fragility, especially in its cortical structure after three months of landing^93^.

With respect to bone remodeling, it is considered that osteocytes are a candidate mechano-sensing cell in mammals, as they are localized in bone tissue and throughout the bone canal^94^. It is expected that specific markers for osteocytes (e.g., sclerostin, a known marker of osteocytes, which are also expressed in osteoblasts^95^) will be found in the near future to clarify the role of osteocytes in response to mechanical stress^95^. With that said, medaka has no osteocytes but still shows altered bone metabolism under microgravity, suggesting the existence of a response mechanism to gravitational stress that does not involve osteocytes.

### Activities in the world

As shown below, interesting researches on muscles and bones are being conducted overseas. Italian Mice Drawer System and Bion-M1 missions have examined changes in the bone architecture of spaceflight mice using high-resolution X-ray tomography^96,97^. The results of these studies clearly show that some (e.g., femur), but not all (e.g., parietal bone), weight-bearing bones (e.g., femurs and VII lumbar ring) are significantly reduced during spaceflight. Furthermore, the US National Aeronautics and Space Administration (NASA) has also sent 40 genetically modified mice to space to evaluate whether blocking the actions of myostatin prevents skeletal muscle atrophy in space^98^. Systemic inhibition of myostatin/activin A signaling using a soluble form of activin type IIB receptor, which can bind both these ligands, led to dramatic increases in both muscle and bone mass in space-flown mice. Eli Lilly and Company performed a similar mouse spaceflight experiment using a myostatin inhibitor that specific antibody-mediated inhibition of myostatin restores skeletal muscle and strength loss caused by microgravity, but not in the soleus muscle^99^. Together, these findings suggest that inhibition of myostatin signaling may be an effective countermeasure against the skeletal muscle and bone loss experienced by astronauts under microgravity.

As a notable achievement, in the recent NASA Twins Study, in which identical twin astronauts were examined before and after a 1-year spaceflight, changes in immune responses, epigenetics, gut microbiota, body weight, and serum metabolites were observed after the astronauts’ return to Earth^66^. In addition, some changes, including a small subset of changes in gene expression, telomere dynamics, DNA disruption, carotid artery thickening, ocular tissues, and some cognitive functions, remained at 6 months after the astronauts’ return to Earth. The risk factors for the persistence of molecular changes (e.g., gene expression) after long-term space missions (>1 year) remain to be elucidated. The results from overseas and JAXA missions have points in common. Accumulating the common points between them will lead to the future development of space biology researches.

### Limitations

Automatic analytical technologies such as single-cell RNA and DNA sequencing, omics analysis, and imaging are advancing day by day. They are also used for detailed analysis of biological samples in space flight. By improving each of these devices so that they can be used under microgravity and installing them at the ISS laboratory, it is possible to immediately study biological phenomena that change in space. It is also expected to reduce the return of in-flight samples for analysis on Earth and significantly reduce the time required for crew members to support experiments in space. Further innovations in these technologies and devices will help clarify the phenomena of life and will bring great progress in the field of space life science.

### Future directions

The ISS has already been developed as a space colony, and plans are now underway to build a gateway to the moon and even to go to Mars, meaning that humans will have to live in space for a long period of time. Recent research has shown that exercise together with bisphosphonate treatment can partially ameliorate the bone loss that occurs in space^100,101^, however, the side effects associated with bisphosphonate are a concern. Understanding more about the mechanisms of gravitational stress at the molecular level will promote the development of small-molecule drugs, such as those that selectively regulate epigenetics or transcription factors^90^, as a means of protecting against muscle atrophy and osteopenia.

Here, we have provided a unique overview of the findings of JAXA missions (Fig. 4). The data presented here are important, as there is a need to maximize information from space data sources from different missions and from different agencies and across research disciplines to ensure maintenance of astronaut health during long-term missions to Mars or beyond^102^. In addition, some of the countermeasures used in spaceflight by astronauts could be used to help in the maintenance of the health of older persons on Earth. Thus, these findings could have an extensive application to improve elderly quality of life, prevent falls, and to ensure active and healthy aging^103^.
